# Design of an Oximeter Based on LED-LED Configuration and FPGA Technology

**DOI:** 10.3390/s130100574

**Published:** 2013-01-04

**Authors:** Radovan Stojanovic, Dejan Karadaglic

**Affiliations:** 1 Faculty of Electrical Engineering, University of Montenegro, Cetinjski put b.b. Podgorica, 81000, Montenegro; 2 School of Engineering and Built Environment, Glasgow Caledonian University, City Campus 70 Cowcaddens Rd., Glasgow G4 0BA, UK; E-Mail: Dejan.Karadaglic@gcu.ac.uk

**Keywords:** photoplethysmography, light emitting diode, sensor, oxygen saturation, FPGA

## Abstract

A fully digital photoplethysmographic (PPG) sensor and actuator has been developed. The sensing circuit uses one Light Emitting Diode (LED) for emitting light into human tissue and one LED for detecting the reflectance light from human tissue. A Field Programmable Gate Array (FPGA) is used to control the LEDs and determine the PPG and Blood Oxygen Saturation (S_p_O_2_). The configurations with two LEDs and four LEDs are developed for measuring PPG signal and Blood Oxygen Saturation (S_p_O_2_). N-LEDs configuration is proposed for multichannel S_p_O_2_ measurements. The approach resulted in better spectral sensitivity, increased and adjustable resolution, reduced noise, small size, low cost and low power consumption.

## 1. Introduction

Photoplethymysography (PPG) is a non-invasive method for the detection of cardiovascular pulse waves propagating across the human body. It is based on the determination of optical properties of vascular tissue using a probe, which consists of LED-photodiode configuration [1,2]. The LEDs run as light emitters and the photodiodes (usually PINs, diode with a wide, lightly doped “near” intrinsic semiconductor region between a p-type semiconductor and an n-type semiconductor region) as light detectors (photo detectors, PDs). The probe can be placed on the periphery of human body, most commonly on a finger or a toe and can operate in reflectance or transmittance mode. The emitted light is reflected, absorbed or scattered by the blood and tissues. The intensity of the light reaching the PD is measured and the variations, caused by blood volume changes, are amplified, filtered and recorded as a voltage signal. This signal is extremely small, subject to noise and in addition to the PD, precise analogue amplifiers, high order filters and analogue-to-digital converters are required [3]. Additional components not only increase the system complexity and cost, but also its size and power consumption. Additionally, the PIN detectors are not ideal as they are not spectrally selective and indiscriminately detect broad spectrum light ranging from near infrared to UV, and all that contributes to the noise and error levels.

**Figure 1.**
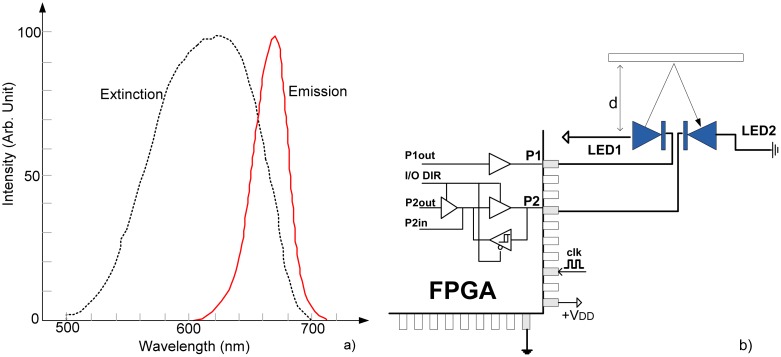
(**a**) Spectral characteristic of LED in emission and extinction (detecting) mode [4]. (**b**) FPGA-based LED-LED distance meter.

On the other hand, it has been neglected that LED in reverse operation mode can also detect light and can be used in a wide range of applications as an inexpensive, readily available optical detector [4–6]. Typically, an LED detects light at a wavelength slightly shorter than the light it emits, making it a wavelength-selective detector, as seen in Figure 1(a) for case of red LED (670 nm) [4]. As such, the LED can be effectively used as an alternative to PIN detector in the case of PPG sensing.

The second challenge is in the fact that, today, numerous miniature, battery powered medical devices tend to be simple, robust and multifunctional [7]. Solution can be achieved by using low-cost sensors and actuator embedded into single platform, preferably System on Chip (SoC), while maintaining necessary accuracy.

Inspired by the possibility to use LEDs as an optical detector as well as by the advantages of nowadays highly integrated chips like FPGA, a PPG sensor requiring only standard LEDs as sensors and FPGA as actuator has been developed. Thus, fully digital, single-chip, solution for multichannel PPG measurement is obtained. 2-LEDs configuration is used for PPG measurement, 4-LEDs configuration for measurement of S_p_O_2_, while N-LEDs system can be employed for multichannel S_p_O_2_ measurements.

Section 2 continues with the explanation of proposed sensing methodology. The implementation of sensing algorithm in FPGA technology is elaborated in Section 3, while Section 4 presents the test results.

## 2. Sensing Methodology

**Figure 2.**
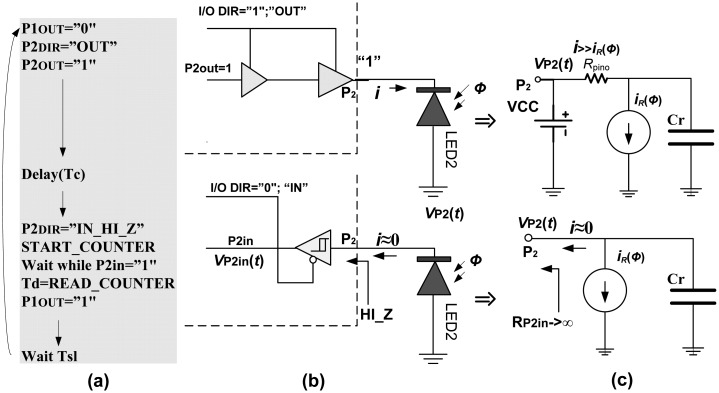
(**a**) Algorithmic steps. (**b**) LED's biasing process. (**c**) Equivalent circuits.

The basic sensing circuit is shown in Figure 1(b). It consists of two infrared LEDs, directly connected to the FPGA pins. Emitter (LED1) is driven by the output pin P1, while receiver (LED2) is connected to the pin P2, which can operate in output or input mode depending on I/O DIR. The light is reflected from an obstacle which is located at distance *d*. The algorithmic steps and electrical models are given in Figure 2. The process starts with switching pin P1 to low state, turning on LED1. Simultaneously, pin P2 is switched to high state, for a very short time *T_c_* (0.1 μs–0.5 μs), charging LED2 to voltage V_DD_ (+5 V or +3.3 V), across very low output impedance *R_pino_*. This charge is held by the sum of the inherent capacitances of the diode and I/O pin itself, *C_r_* = *C_rLED_* + *C_rpin_*. Typically *C_rLED_* = 10 pF–15 pF and *C_rpin_* = 3 pF–5 pF. Then, pin P2 is switched into high-impedance (Hi-Z) mode, approximately 10^15^ Ώ, and digital counter/timer starts.

Under reverse bias conditions and high impedance of pin P2, the LED2 acts as a capacitor *C_r_* discharged by a current source *i*_R_(*Φ*), which models the optically induced photocurrent for incoming light intensity *Φ*. The process of *C_r_* discharging, assuming that *i*_R_(*Φ*) remains constant during short time, can be expressed as:

(1) $$\nu_{P2}(t)=V_{\text{CC}}-\frac{1}{C_{r}}\int_{o}^{t}i_{R}(t)\text{dt}=\text{Vcc}-\frac{i_{R}\left(\Phi \right)}{C_{r}}t$$

that means *ν*_P2_(*t*) linearly decreases with time *t* to zero. During discharging *ν*_P2_(*t*) is continually polled through its digital equivalent P2in. When P2in changes from logical “1” to “0”, *ν*_P2_(*t*) falls below the threshold voltage *V_TR_*. For CMOS FPGA devices V_TR_ is about $\frac{V_{\text{DD}}}{2}$. Immediately, the counter/timer is stopped and the decay time (*Td*) is read as a number of clock ticks *N*:

(2) $$T_{d}[\text{ms}]=\frac{C_{r}}{i_{R}\left(\Phi \right)}\left(V_{\text{CC}}-V_{\text{TR}}\right)=NT_{\text{tclk}}=N\frac{1}{f_{\text{tclk}}}$$

where *f_tclk_* presents frequency of timer/counter clock.

**Figure 3.**
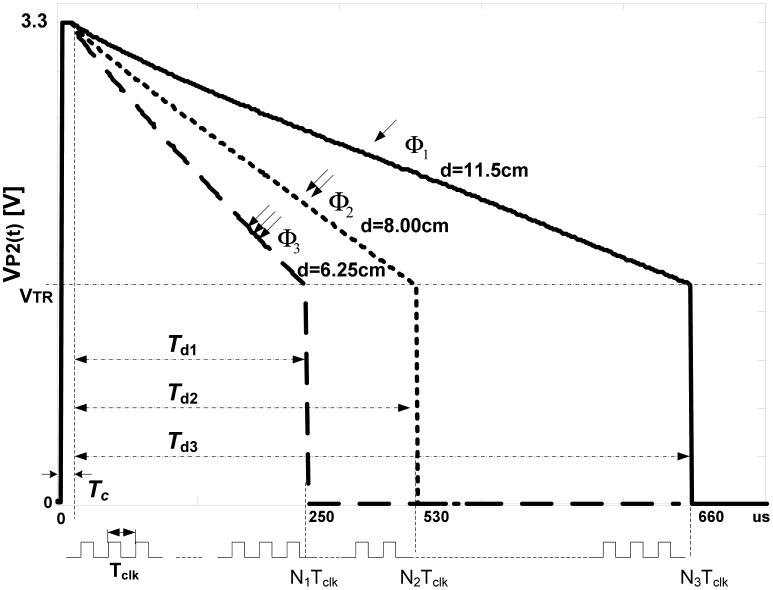
The processes of LED discharging for different *Φ_i_*, with corresponding decay times *T_di_*.

Experimentally recorded *ν*_P2_(*t*) curves, obtained through this project, follow Equation (1) that is illustrated in Figure 3 for three levels of light fluxes, *Φ_1_*, *Φ_2_* and *Φ_3_*, *Φ_1_* < *Φ_2_* < *Φ_3_*. It is apparent that *T_d_* decreases when the amount of received light increases and vice versa. For light intensities *Φ_1_*, *Φ_2_* and *Φ_3_*, *N* corresponds to *N_1_*, *N_2_* and *N_3_*. The resolution of *T_d_* can be adjusted by *f_tclk_* ¸ taking care about overflow, 2*^l^* − 1 < *N_max_*, *l* is counter length. Usually, *f_tclk_* is obtained from main clock *f_clk_* by frequency divider with selectable prescale factor *N_p_*, *f_tclk_* = *f_clk_/N_p_*, *N_p_* = 1, 2, 4, 8, 16…. This gives a possibility to adjust resolution of *T_d_*, marked as *Δ*T_d_, with prescaler *N_p_* as:

(3) $$T_{d}=N\frac{N_{p}}{f_{\text{clk}}}=N\;\Delta \;T_{d}$$

As example, for *f_clk_* = 200 MHz and *N_p_* =*1*, *ΔT_d_* can reach value of 5 *ns*. The sensing cycle repeats with frequency *f_sl_* = *1*/*T_sl_*, which is in fact the sampling frequency of PPG signal.

**Figure 4.**
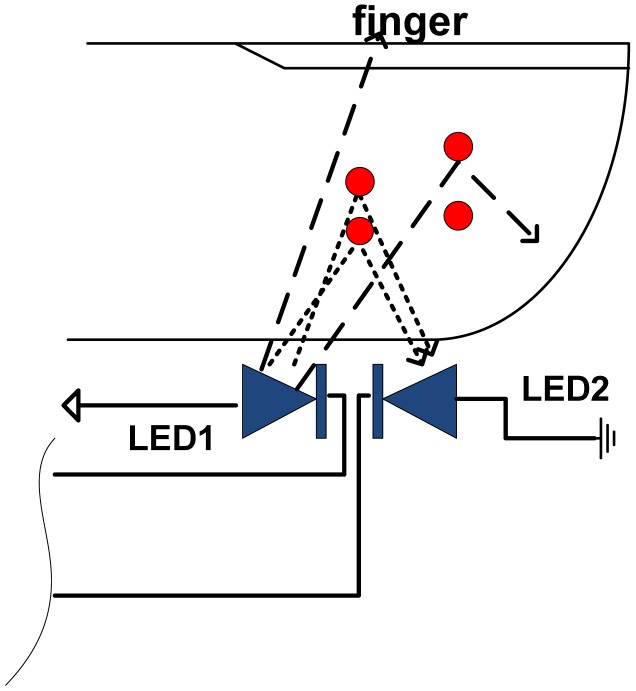
Blood fluctuation sensing using two LEDs.

In the case of human finger, Figure 4, or toe, due to the configuration of light paths, the photo current is proportional to the volume and the fluctuations of blood inside the finger or lobe. It is in the range of 10^−12^A (1 pA) in total darkness to about 10^−6^A (1 μA) under maximal reflectance. The blood cells (especially reds) play role of obstacle. Performed experiments with volunteers shown that for *C_r_* = 15 pF and *V_TR_* = *V_DD_*/2, *T_d_* varies from 0.1 ms to 1 ms that corresponds to the counter resolution of above 2^10^ to 2^15^.

The LED-LED sensing probe is of low energy consumption. If *I*_CCH_ and *I*_CCL_ are maximal currents of FPGA's O/I pins during their high and low states, *T*_sl_ ≫ *T*_d_, *T*_d_ ≫ *T*_c_, then power dissipation of 2-LEDs system is given as:

(4) $$P_{2-\text{LEDs}}=P_{\text{LED}1}+P_{\text{LED}2}=V_{\text{CC}}\frac{1}{T_{\text{sl}}}\int_{0}^{\text{Tsl}}I_{\text{CCl}}\text{dt}+\frac{1}{C_{r}}\int_{0}^{\text{Tsl}}I_{\text{CCH}}\text{dt}=I_{\text{CCL}}\frac{T_{d}+T_{C}}{T_{\text{sl}}}+V_{\text{CC}}I_{\text{CCH}}\frac{T_{C}}{T_{\text{sl}}}≅V_{\text{CC}}I_{\text{CCL}}\frac{T_{d}}{T_{\text{sl}}}$$

As an example, for *V*_DD_ = 3.3 V, *I*_CCH_ ≈ *I*_CCL_ = 25 mA, *T*_d_ = 0.15 ms and *T*_sl_ = 10 ms (*f*_sl_ = 100 Hz), the dissipation of LED1-LED2 system is about 1.2 mW, while for *T*_sl_ = 100 ms (*f*_sl_ = 10 Hz) it decreases to 0.12 mW.

**Figure 5.**
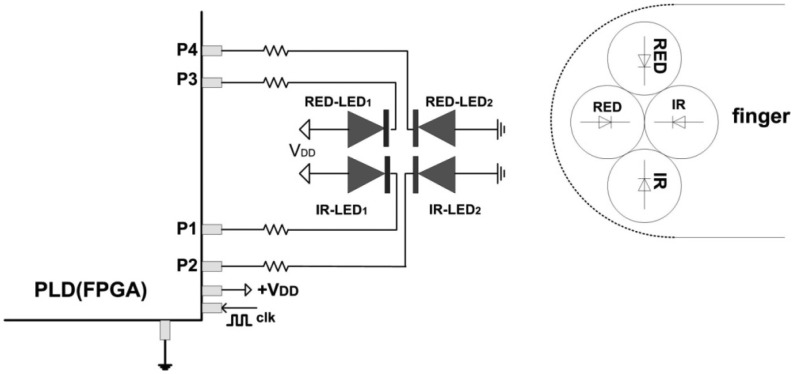
FPGA-based S_p_O_2_ meter.

The PPG configuration from Figure 4 can be easily expanded to include a S_p_O_2_ meter, by adding two more red LEDs (Figure 5). Light from LEDs at two different wavelengths is transmitted through the tissue bed and the PD measures the unabsorbed light. The method exploits the fact that deoxyhaemoglobin (Hb) has higher optical extinction in the red region (R) of spectrum than oxyhaemoglobin (HbO_2_) and lower optical absorption in the near infrared region (IR) [8]. These differences in the extinction coefficients can be used for the determination of the light absorbed by Hb and HbO_2_, and then used to calculate the “*normalised ratio*”:

(5) $$R=\frac{R}{\text{IR}}=\frac{{\text{AC}}_{R}\;/\;{\text{DC}}_{R}}{{\text{AC}}_{\text{IR}}\;/\;{\text{DC}}_{\text{IR}}}$$

which correlates to S_p_O_2_ according to empiric equation:

(6) $$S_{p}O_{2}=110-25R≅110-25\frac{\frac{\text{RMS}(\text{RED})}{\text{MEAN}(\text{RED})}}{\frac{\text{RMS}(\text{IR})}{\text{MEAN}(\text{IR})}}$$

where MEAN represents DC component calculated by averaging the signals over a time, RMS AC component, calculated by averaging the square of the signal over a time. The AC component is divided by the DC component in order to eliminate the influence of time invariant absorbance from venous blood or surrounding tissues.

Knowledge of S_p_O_2_ can assist in the diagnosis of various respiratory conditions and ischemic states. Normal value is from 95%–100%. A smokers could have values as low as 90% and someone with Chronic Obstructive Pulmonary Disease (COPD) <85% [9].

Sensing algorithm for S_p_O_2_ measurements is practically double algorithm from Figure 2, adding two more red LEDs, which are driven by pins P3 and P4. Two decay times *T*_d_ = *T*_dred_ and *T*_d_ = *T*_dir_ are measured in parallel with separated timers/counters. The power consumption is also double, but does not exceed 2.5 mW.

## 3. FPGA Implementation

Previously described PPG and S_p_O_2_ detection algorithms could be implemented by using general-purpose microcontrollers (MCs) or PLDs. Designers often choose MCs because they traditionally offer better on-chip peripherals, such as timers, ADCs, UARTs, pulse width modulation (PWM) and other integrated I/O for multiple, asynchronous tasks [3,10,11]. Because they are of general purpose, MCs are more flexible and less specialized, which usually makes them easier for understanding and programming. Using MCs in this case is easier and more economical way, but there are several restrictions such as Von Neumann (sequential) architecture, limited numbers of fixed length timers/counters, clock frequency bellow 16 MHz, low DSP capabilities, insufficient parallelism, several software layers *etc.*

Unlike processors, FPGAs use dedicated hardware for processing logic and do not have an operating system (OS). Since FPGA are there truly parallel in nature, different processing operations do not have to compete for the same resources. FPGAs and processors also differ in compilation. When an application for an FPGA device is compiled, the result is a highly optimized silicon implementation that provides parallel processing with the performance and reliability benefits of dedicated hardware circuitry. Because there is no OS on the FPGA, the code is implemented in a way that ensures maximum performance and reliability. In contrast to early families, nowadays FPGAs do not only offer a lot of logic base cells, but also huge register blocks and memory areas and powerful processor cores, which speed may achieve 500 MHz.

Considering experience and results obtained during development and investigation of MC based PPG sensors [6], this project migrated to FPGA. There are few attempts to use FPGA for PPG signal detection, like one in [12]. It still uses PIN diode as PD and do not have possibility to measure S_p_O_2_ and to perform on-line signal processing.

**Figure 6.**
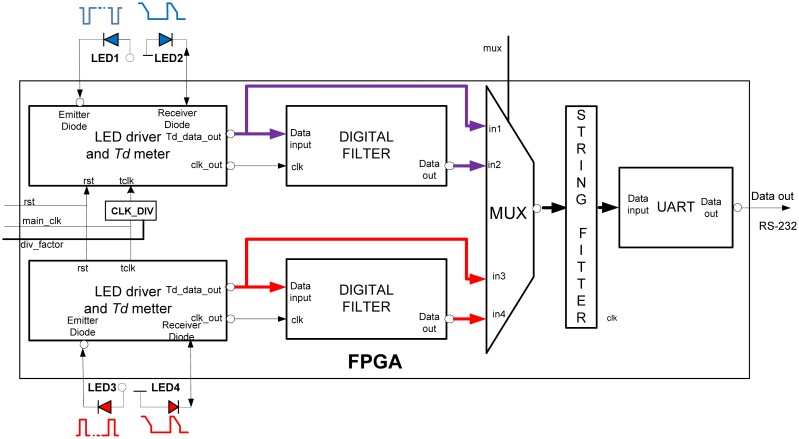
FPGA implementation of PPG and S_p_O_2_ sensor/actuator.

The FPGA architecture, proposed in this paper, consists of several functional units, Figure 6. Two identical *LED drivers* and *Td meters* simultaneously excite emitter diodes LED1and LED3 and simultaneously measure the decay times *T_dir_* and *T_r_* (IR and RED) by tracking voltages on LED2 and LED4. Note that in case of classical pulse oxymeters the time multiplex is used to distinguish between the signals from different light sources. Such multiplex is not necessary here because the LED1, LED3 and LED2, LED4 are switched on and read in parallel. The digital equivalents of *T_dir_* and *T_r_* in form of counter/timer values, are sent to the *UART* directly or via a *Digital filter* section, depending on the MUX selection. *CLK_DIV* determines counter frequency *f_tclk_* for different prescaled factors. *String fitter* (SF) packs the samples in predefined order, IR-RED…-IR-RED, while *UART* sends the original or filtered data to the host computer in selectable speeds. All functional modules are realised in form of VHDL code functions with generic arguments (data widths, filter order, sampling frequency, communication speed, pipeline and so on).

In order to optimize the design and improve Signal to Noise Ratio (SNR) we investigated several types of digital filters like *Mean*, *FIR*, *and IIR*. The filter function is implemented through the transposed structure which handles all types:

(7) $$Y(z)=c\frac{b(1)+b(2)z^{-1}+\ldots +b(\text{nb}+1)z^{-\text{nb}}}{1+a(2)z^{-1}+\ldots +a(\text{na}+1)z^{-\text{na}}}X(z)$$

where *n-1* is the filter order, *n_a_* is the feedback filter order, and *n_b_* is the feedforward filter order. As example, for *c* = 1/N and *b(1)* = *0, b(2)* = … = *b(nb* + *1)* = *1, a(1)* = *a(2)* = … = *a(na* + *1)* = *0* it becomes mean filter and for *c* = *1, a(1)* = *a(2)* = … = *a(na* + *1)* = *0* symmetric FIR, *etc.* Coefficients *a(i)*, *b(i)* and *c* are multiplied and rounded in order to be pre-scaled in integer arithmetic [13].

**Figure 7.**
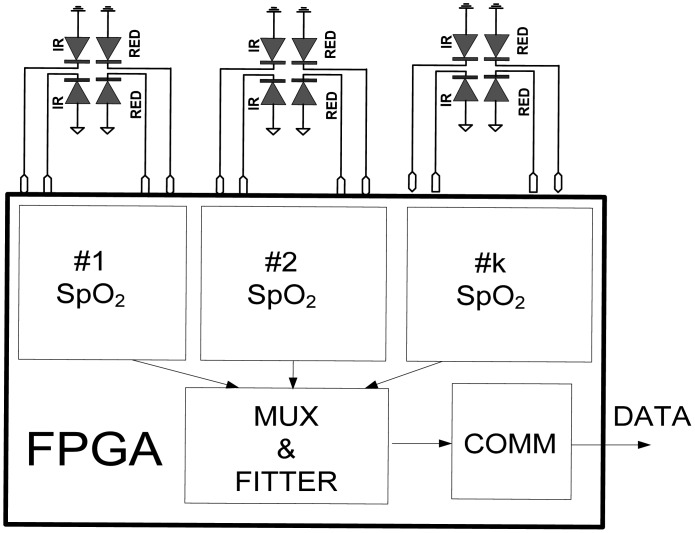
Multichannel S_p_O_2_ system implanted in a single FPGA chip.

Today, physiology and cardiology is facing the challenge of performing pulse oximetry measurements at other body locations where arterial blood perfusion is severely degraded. Low and heterogeneous perfusion conditions can be overcome by a multi-channel oximetry approach that simultaneously exploits spatial, frequencies and temporal diversities. The proposed LED-LED FPGA approach is very suitable for such applications, as is shown in Figure 7. Several S_p_O_2_ probes can be connected to a single FPGA chip and processed in parallel.

## 4. Results

The proposed PPG sensor-actuator was tested in both PPG and S_p_O_2_ configurations against qualitative and quantitative criteria. In PPG configuration, the sensing probe comprises an IR(910 nm)-IR(910 nm) 5 mm LED combination and the signal is collected in reflectance mode from a finger. As a substrate, an FPGA chip from Altera, Cyclone III, series, EP3C10 was chosen (device EP3C10E144C8) [14]. The inherent capacitance of its I/O pin is about 4 pF. The I/O pins are configured for 3.3 V operation. Running clock was 48 MHz, allowing maximal resolution of 20 ns, towards temperature of 30 °C. The signals are sampled at 100 Hz or 200 Hz and sent via RS232 interface at 19,200 bps to host computer, where a MATLAB-based virtual instrument is designed for data importing, post-processing and displaying. The virtual instrument allows additional filtering, Fast Fourier Transform (FFT), Time-Frequency Analysis (TFA), statistics, *etc.*, all in real time.

**Figure 8.**
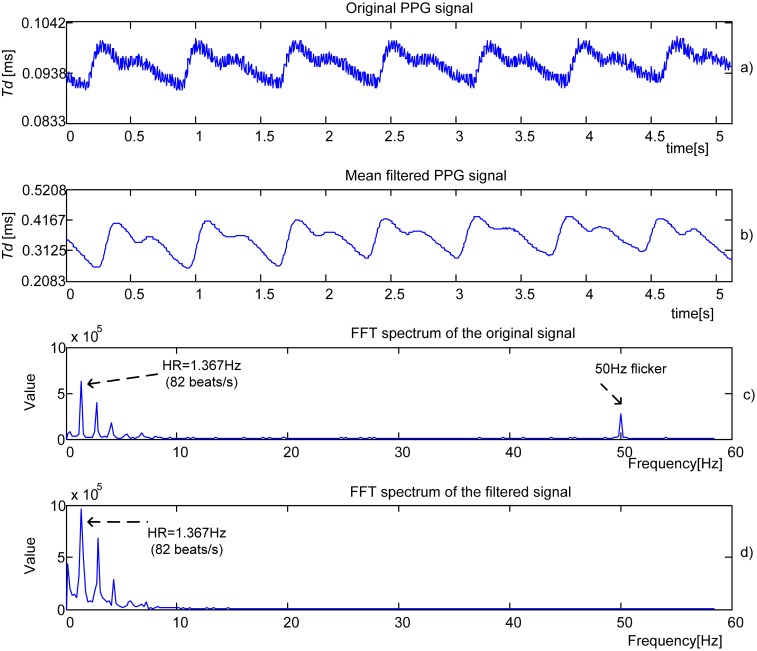
Experimental PPG signal (**a**) and signals obtained after processing by *16th order Mean filter* (**b**) and FFT, (**c**) and (**d**), *f*_sl_ = 200 Hz.

Figure 8(a) shows the original time domain PPG signal obtained by IR-IR combination from a 23-year old male student. The filtered component obtained by 16th order *Mean filter* is shown in Figure 8(b). The Fourier spectrum of the original signal is shown in Figure 8(c). The heart rate (HR) frequency corresponds to the dominant peak. The frequency that represents a flicker, 50 Hz noise induced from mains or room lights, is also visible. Figure 8(d) shows the Fourier spectrum of the filtered signal. The process parameters are *f_tclk_* = 48 MHz, *N_p_* = 1 and *f*_sl_ = 200 Hz.

**Figure 9.**
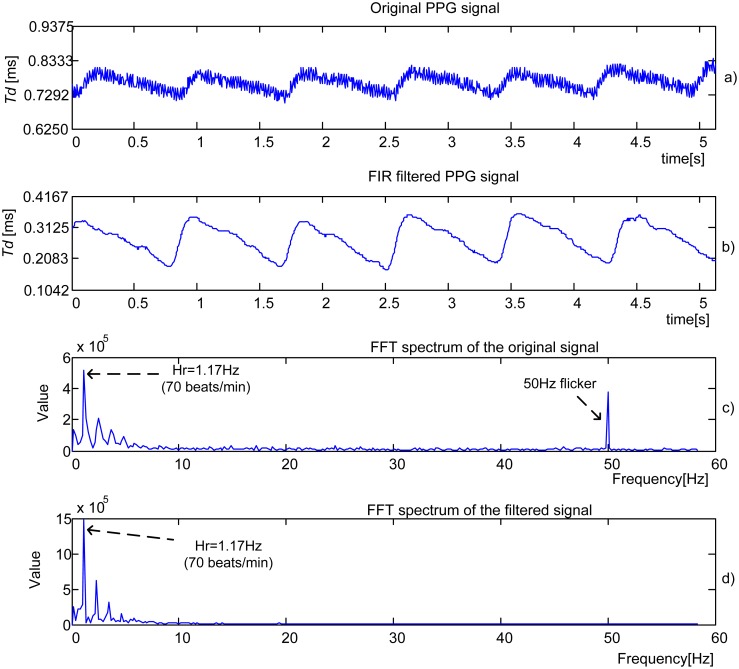
Experimental PPG signal (**a**) and signals obtained after processing by 16th order *low pass FIR filter* (**b**) and FFT, (**c**) and (d), *f*_sl_ = 200 Hz, *f*_c_ = 10 Hz.

Figure 9(a) presents an original PPG signal recorded from a 22-year old female student. The signal is processed by 16th order *low pass FIR* filter with cut-off frequency *f*_c_ = 10 Hz and *f*_sl_ = 200 Hz, producing an output given in Figure 9(b). Follow the Fourier spectrums of the original and filtered signals, Figure 9(c) and Figure 9(d). Again HR is detected as a dominant peak.

**Figure 10.**
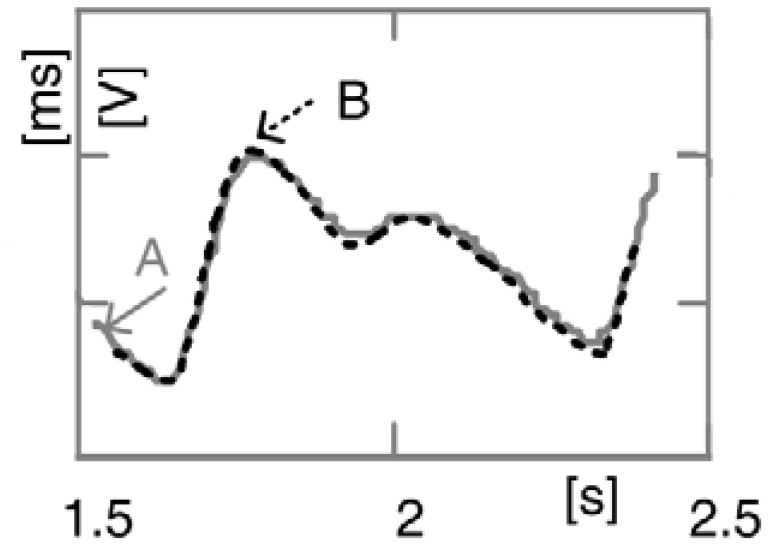
Comparison of obtained signals, (**A**) proposed, (**B**) Nonin's system (8000AA sensor + OEM III Eval Kit + Software).

Qualitative comparison between proposed sensor (A) and its conventional FDA approved equivalent (B), *Nonin Model 8000AA* [15] *(Adult Articulated Finger Clip)*, is presented in Figure 10. The signal indicated by dashed line was recorded by model 8000AA. As seen, these two signals match almost perfectly.

**Figure 11.**
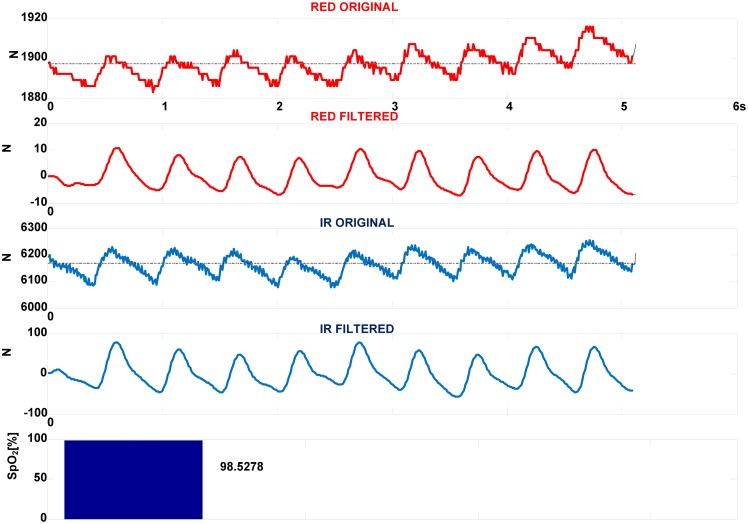
Calculation of S_p_O_2_ from PPG signal using 4-LEDs configuration in conjunction with FPGA. The signal is filtered by 3rd order *low-pass Butterworth* filter, *f_sl_* = 100 Hz, *f*_c_ = 10 Hz.

Figure 11 presents IR(910 nm) and RED(660 nm) components of PPG signals obtained by 4-LEDs in, S_p_O_2_ configuration, *f_sl_* = 100 Hz, *f*_tclk_ = 16 MHz, *N_p_* = 3. The original signals are filtered by 3rd order *low-pass Butterworth filter* with border frequency of *f*_c_ = 10 Hz. As seen, the original signals contain large DC components or offsets. Superimposed to them are AC components reflecting the pulsatile component of circulation that theoretically can vary between 0.1% and 1% of the DC level. The corresponding MEAN and RMS values are MEAN(RED) = 1,896.95, RMS(RED) = 4.96, MEAN(IR) = 6,165.14, RMS(IR) = 35.14. Using Equation (6) the S_p_O_2_ is calculated to 98.53%.

Quantitative testing was performed considering following criteria: occupation of silicon resources for hardware implementation in different configurations, comparison of accuracy PPG and S_p_O_2_ measurement against clinical reference and maximal average dissipation for 2-LEDs and 4-LEDs configurations.

Table 1 illustrates the occupancy of silicon resources on target devices (EP3C10 with 10,320 LCs—Logic Cells) for different combinations of built in components. As seen, the FPGA chip of higher capacity can serve several S_p_O_2_ instruments. It is very useful for multi-point blood saturation examination. As example in EP3C120 family about 30 S_p_O_2_ instruments in configuration 4 LEDs + UART + MUX + ST + FIR F. can be implanted.

The testing experiments for PPG and S_p_O_2_ accuracy are performed on volunteers of different age and health conditions. About 120 different subjects were examined. The measured value of %S_p_O_2_ and %HR were compared with Nonin's system, 8000AA sensor + OEM III Eval Kit + Software [14]. The experimental values are shown in Table 2.

Additionally, two systems are compared in term of maximal average power consumption. The results are given in Table 3. Although 8000AA is a low power sensor, the proposed solution has lower consumption. A 4-LEDs sensor with a EP3C10 FPGA system has 48 mW consumption that is slightly more than 36 mW for Nonin's solution, but in case of two S_p_O_2_ instruments in a single FPGA, the consumption falls to 25 mW per instrument that is better than in Nonin's case.

Obviously, the proposed system is a low cost taking in account prices of LEDs (less than 0.5$ per piece) and FPGA chip (about 15$). The cost of experimental system, presented here, was about 50$, six times less than Nonin's equivalent 8000AA sensor + OEM III Eval Kit.

## 5. Conclusions

We have demonstrated the possibility to design an effective system for measurement of PPG signals and blood oxygen saturation by using a LED-LED based configuration in conjunction with a FPGA chip. The proposed sensing technique is fully digital and provides good spectral sensitivity, increased resolution, satisfactory signal to noise ratio, low power consumption, in system flexibility and reconfigurability and miniaturisation. By using the presented approach the challenges of multi-channel oximetry can be overcome.

## Figures and Tables

**Table 1.** FPGA resources for different configurations of PPG sensor, expressed in LCs.

**Configuration**	**# of LCs**	**Occupancy in LCs [%], EP3C10**
2 LEDs + UART + MUX + ST	311	3%
2 LEDs + UART + MUX + ST + MEAN F.	861	8.3%
2 LEDs + UART + MUX + ST + FIR F.	1,866	18.1%
4 LEDs + UART + MUX + ST	634	6.1%
4 LEDs + UART + MUX + ST + MEAN F.	1,746	17%
4 LEDs + UART + MUX + ST + FIR F.	3,833	37.1%

**Table 2.** Accuracy against industrial reference.

**# of samples**	**Mean absolute error of [%] HR**	**Mean absolute error in [%] S_p_O_2_**
120	0.75	1.2

**Table 3.** Comparison of power consumption.

**Solution/Configuration**	**Consumption**
Nonin's 8000AA-2LEDs	3 mW
Nonin's 8000AA-2LEDs	6 mW
Proposed-2LEDs	1.5 mW
Proposed-4LEDs	3 mW
Nonin's 8000AA sensor + OEM III Eval Kit	36 mW
Proposed 4-LEDs sensor + EP3C10 FPGA	48 mW
Proposed 2 × 4-LEDs sensor + EP3C10 FPGA	54 mW, 27 mW per instrument
